# School-based diagnosis and treatment of malaria by teachers using rapid diagnostic tests and artemisinin-based combination therapy: experiences and perceptions of users and implementers of the *Learner Treatment Kit*, southern Malawi

**DOI:** 10.1186/s12936-017-1964-z

**Published:** 2017-08-07

**Authors:** Treza Mphwatiwa, Stefan Witek-McManus, Austin Mtali, George Okello, Paul Nguluwe, Hard Chatsika, Natalie Roschnik, Katherine E. Halliday, Simon J. Brooker, Don P. Mathanga

**Affiliations:** 1 Department of Community Health, College of Medicine, Blantyre, Malawi; 2 London School of Hygiene and Tropical Medicine, London, UK; 3 Save the Children International, Zomba, Malawi; 4 KEMRI Wellcome Trust Research Programme, Kilifi, Kenya; 5 Save the Children, USA, Westport, Connecticut USA; 6 Malaria Alert Centre, College of Medicine, Blantyre, Malawi

## Abstract

**Background:**

Training teachers to diagnose uncomplicated malaria using malaria rapid diagnostic tests and treat with artemisinin-based combination therapy has the potential to improve the access of primary school children (6–14 years) to prompt and efficient treatment for malaria, but little is known about the acceptability of such an intervention. This qualitative study explored experiences and perceptions of users and implementers of a programme of school-based malaria case management via a first-aid kit—the Learner Treatment Kit (LTK)—implemented as part of a cluster-randomized controlled trial in Zomba district, Malawi.

**Methods:**

From 29 primary schools where teachers were trained to test and treat school children for malaria using the LTK, six schools were purposively selected on the basis of relative intervention usage (low, medium or high); school size and geographical location. In total eight focus group discussions were held with school children, parents and guardians, and teachers; and 20 in-depth interviews were conducted with key stakeholders at the school, district and national levels. Interviews were recorded, transcribed, and analysed using a thematic analysis approach.

**Results:**

The LTK was widely perceived by respondents to be a worthwhile intervention, with the opinion that trained teachers were trusted providers of malaria testing and treatment to school children. Benefits of the programme included a perception of improved access to malaria treatment for school children; decreased school absenteeism; and that the programme supported broader national health and education policies. Potential barriers to successful implementation expressed included increased teacher workloads, a feeling of inadequate supervision from health workers, lack of incentives and concerns for the sustainability of the programme regarding the supply of drugs and commodities.

**Conclusion:**

Training teachers to test for and treat uncomplicated malaria in schools was well received by both users and implementers alike, and was perceived by the majority of stakeholders to be a valuable programme. Factors raised as critical to the success of such a programme included ensuring an effective supervisory system, a reliable supply chain, and the training of greater numbers of teachers per school to manage high consultation numbers, especially during the peak malaria transmission season.

## Background

Malaria among primary school-age children has long been recognized as a threat to the health and education of this age group, but has attracted relatively little attention [1]. Recent surveys conducted across sub-Saharan Africa (SSA) have reported that the prevalence of *Plasmodium falciparum* in school-age children is consistently higher than all other age groups, but has significant variation across the region—from 4% of children in western Tanzania [2] to 60% or higher in Malawi and southern Mali [3]. In Malawi, primary school-aged children are at higher risk of *Plasmodium* infection than younger children, but their access to malaria prevention and formal case management services is lower than other age groups [4, 5]. The high prevalence of asymptomatic infection and associated morbidity has the potential for detrimental effects on educational achievement, through reduced school attendance and cognitive development [6, 7]. However, given the rapid increase in primary school participation across SSA [8], schools increasingly have the potential to be an efficient and effective delivery point to provide care for malaria.

Previous research has demonstrated that the delivery of anthelmintic treatment by school teachers is widely accepted by both parents and guardians, and school children, and that teachers view this additional role positively [9, 10]. Prior to recommendations that parasitological diagnosis (by rapid diagnostic test or microscopy) should be undertaken before artemisinin-based combination therapy (ACT) is given, past studies have also indicated that using trained school teachers in the provision of presumptive malaria treatment using chloroquine or sulfadoxine-pyrimethamine was feasible [11–13]. A study in coastal Kenya reported that community members would be satisfied with teachers overseeing the delivery of ACT treatments to primary school children following rapid diagnostic tests (RDTs) carried out by health workers, and that they would also be accepting of the teachers conducting RDTs following thorough training [14].

The Ministry of Health (MoH) in Malawi has recently explored the use of RDTs for community case management of malaria in children under 5 years of age [15]. With support from the Ministry of Education Science and Technology (MoEST), the MoH has been keen to expand a ‘test, treat and track’ service to children over 5 years of age, through school-based case management. As a result, the MoH and MoEST established a trial to train teachers to use malaria RDTs and ACT for the diagnosis and treatment of uncomplicated malaria in school children. A pre-implementation pilot workshop in June 2013 indicated that training school teachers to conduct RDTs and treat uncomplicated malaria as part of a basic first aid kit was feasible. Post-training interviews with participants (school teachers) and facilitators (district-, zonal- and national-level facilitators from the MoH) suggested that following sufficient training and practice, teachers felt confident to conduct RDTs and make decisions regarding treatment [16].

### The Learner Treatment Kit

This qualitative study was conducted within the context of a cluster randomized trial involving 58 primary schools in Traditional Authority (TA) Chikowi, Zomba district between 2011 and 2015 where the feasibility, cost-effectiveness and impact of using teachers to diagnose and treat primary school children with uncomplicated malaria was evaluated [16]. Twenty-nine schools received the intervention called the ‘Learner Treatment Kit’ (LTK) between November 2013 and April 2015. The LTK is a basic first-aid kit, available to all primary school children (known in Malawi as ‘learners’) during school hours for the management of basic health problems such as diarrhoea, eye infections, minor wounds, and uncomplicated malaria. The LTK was implemented by the National Malaria Control Programme (NMCP) within the MoH and the School Health and Nutrition Programme within the MoEST; with support from Save the Children in training, supervision, logistical coordination and procurement of drugs and supplies. Prior to implementation, a series of community sensitization meetings were conducted at each intervention school with school staff, caregivers and school children to explain the purpose and scope of the intervention. At these meetings, it was emphasized to caregivers that the intervention was not a replacement for routine health facility-based care but an additional service that could be accessed during school hours. Furthermore, all school children attending an intervention school were encouraged to report to the LTK dispensers when they felt unwell. At least two teachers per school were trained as ‘LTK dispensers’ for 7 days, followed by a 3-day mentorship period at a local health centre. The drugs and supplies for the LTK were distributed through the local government health facility, by way of a written request from the LTK dispenser when they required more supplies. In contrast, no such service was available in control schools where primary school children with symptoms of malaria or other basic illnesses were advised to continue to seek treatment at their nearest health facility.

In this study, the authors present perceptions of both users (school children and indirectly their parents and guardians) and providers (trained teachers or ‘LTK dispensers’) of the LTK, as well as of key stakeholders (also providers), at the community, district and national levels, on the acceptability of school-based malaria case management following a year of programme implementation.

## Methods

### Study site and population

This qualitative study was conducted in TA Chikowi, Zomba District,Malawi. The area covers approximately 495 km^2^, with the Mang’anja, Yao and Lomwe the main ethnic groups. The primary source of employment (68%) in the area is farming. Malaria transmission is perennial, with 60 and 32% of primary school-aged children having malaria parasites and anaemia, respectively, during the peak malaria transmission season [3]. Despite perennial transmission, the school terms can broadly be correlated with various transmission seasons, with first term coinciding with the peak rainy season (January–March), second term occurring during the post-rainy season (May–July) and third term covering the dry season (September–November).

### Sampling procedures

**Fig. 1:**
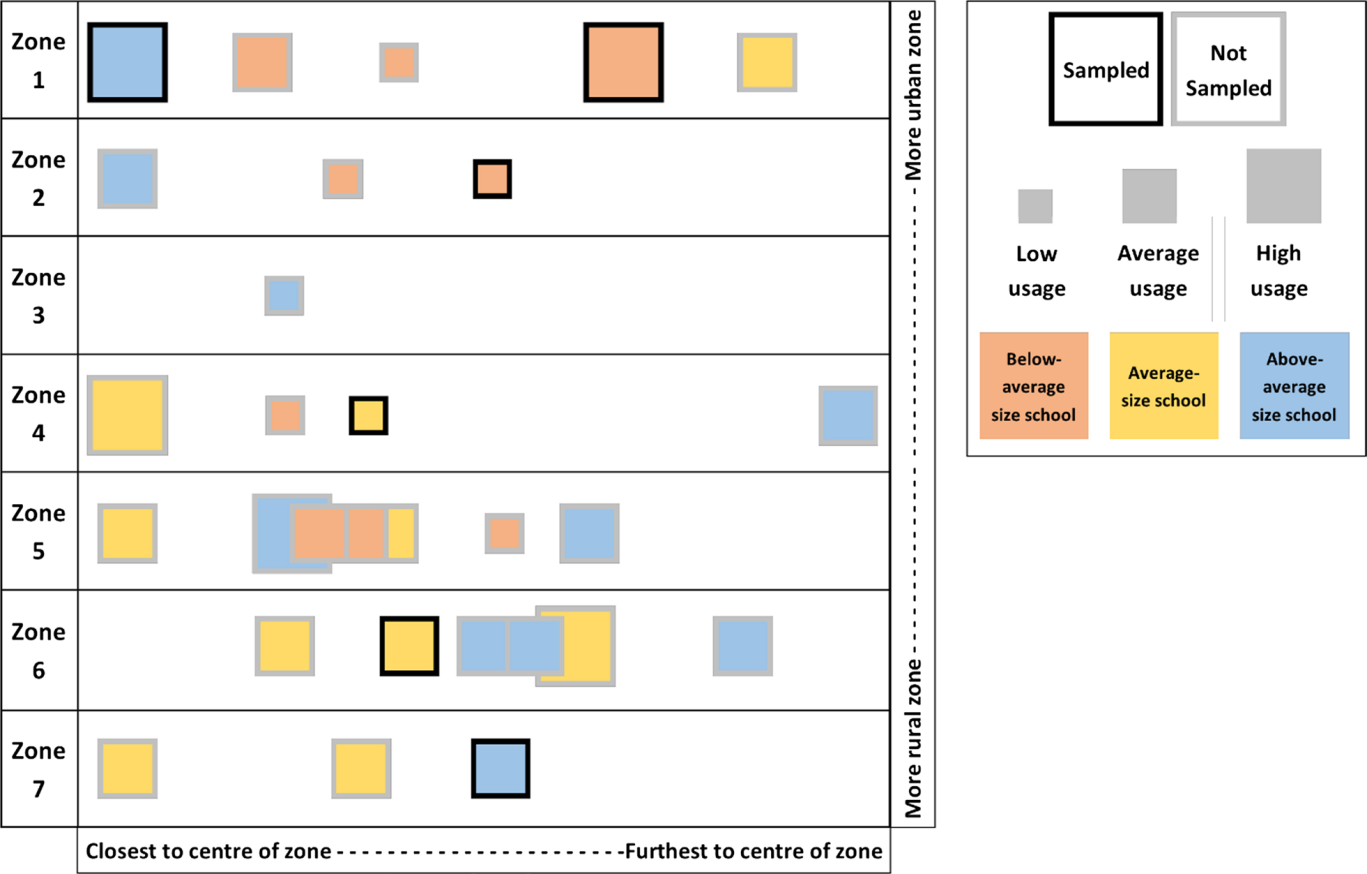
Sampling frame showing the distribution of schools. Diagrammatic representation of LTK intervention schools and their usage of the LTK. Schools are grouped into administrative units known as ‘zones’, which are ordered here by their proximity to the nearest urban area (Zomba city). Schools are shown as a square within each zone and are ordered left to right by straight-line distance from the centre of the zone. The size of each school corresponds to the total number of consultations conducted within the same time period

Purposive sampling was used to select 6 of the 29 intervention schools on the basis of their uptake of the LTK. To capture a range of views on the experiences and perceptions across schools, the relative number of LTK consultations to school size was used as a proxy for uptake. Two schools in each uptake strata (low, medium or high) were selected to take part in the qualitative study. The six schools were also selected to represent a range of school administrative zones (six of the seven zones were covered), school size, and distance from trading centres to allow a wide range of experiences regarding access to care (see Fig. 1). Data collection took place between December 2014 and May 2015.

### Focus group discussions

A total of eight FGDs were conducted with school teachers, parents and guardians and school children from the six primary schools (Table 1). Two FGDs were conducted with teachers. The groups were made up of teachers trained to test and treat malaria (LTK dispensers), as well as teachers who were not trained as part of the LTK, but worked at a school where the intervention was being implemented. Teachers from across the six schools were combined in these FGDs and only teachers who had worked at the school for at least 6 months were eligible to participate. A further three FGDs were conducted with parents and guardians. Each of these three FGDs were conducted in a school from each of the three uptake categories and only contained participants from that single school. To minimize recall bias, eligibility for the parent and guardian FGD was made by identifying children who had sought care from an LTK dispenser in the previous 6 weeks, and inviting their parent or guardian to participate. Finally, three FGDs were conducted with school children involving those that had sought care from the LTK and had been tested and treated for malaria in the 6 weeks prior to the FGD. Again, each of these three FGDs were conducted in a school from each of the three uptake categories and only contained participants from that single school. The FGDs targeted children enrolled in senior classes of Standard 6, 7 or 8 (age range of approximately 11–15 years).

**Table 1:** Types of interviews conducted and number of participants per interview

FGD	Intervention-trained “*LTK Dispenser*” teachers	1 group (n = 12)
FGD	Non-intervention-trained teachers	1 group (n = 12)
FGD	Parents and guardians of children in Standard 6–8 who sought care from the intervention within the past 6 weeks	3 groups (n = 36)
FGD	Children in Standard 6–8 who sought care from the intervention within the past 6 weeks	3 groups (n = 36)
IDI	Intervention school head teachers	n = 6
IDI	Intervention-linked health centre “*in*-*charge*” managers	n = 2
IDI	District level government administrators	n = 6
IDI	National-level government administrators	n = 4
IDI	National-level INGO managers	n = 2

Each FGD comprised of 6–12 participants, a mix of male and female participants, and were conducted in the local language (parents and guardians, and school children FGDs) or English (teachers FGDs). The FGDs were led by the lead author (TM), while two research assistants tape-recorded the discussions and took notes. FGDs were conducted in schools for parents and guardians, and centrally located venues known as a Teacher Development Centre (TDC) for teachers. Participants were provided with refreshments and the equivalent of $1 transport reimbursement for parents and guardians, or $2 for teachers. FGDs lasted approximately 1 h, with discussion topics in the FGD guide including perceptions of malaria, experiences with the LTK, perceptions of the LTK as delivered by teachers—the LTK dispensers, and challenges implementing the LTK. Participants who consented to participate in FGDs were assigned numbers which they stated, instead of their name, prior to commenting or answering questions during the FGDs. This use of identification numbers enabled attribution of perceptions to individuals during coding while also ensuring participant confidentiality.

### In-depth interviews

IDIs were conducted with key stakeholders purposively selected at community, district and national levels. At the community level, six participants were head teachers (one from each of the selected schools) and four participants were healthcare workers from four health facilities that had been orientated on the LTK programme to provide support in terms of mentorship and storing drugs and supplies for the schools. At the district level, participants came from both the district health and education offices. At the national level, participants were senior individuals from the NMCP, MoEST, the National Health Technical Support Services (HTSS) for diagnostics, in addition to a single representative of development partners in Malawi. An IDI guide covering the following thematic areas was used to conduct these interviews: participants’ experiences of implementing school health programmes, and expected roles/challenges in a future roll-out of school-based malaria programmes such as the LTK. Interviews lasted approximately one to one-and-a-half hours each and were held at a time and location that was convenient to participants, most commonly in an office at their place of work.

### Data analysis

Interviews were recorded using audio recorders during the FGDs and IDIs with the permission of participants. All discussions and individual interviews were transcribed verbatim and if required translated from the language conducted (Chichewa) to English. Interview transcripts were imported into NVivo 11 (QSR International Pty Ltd, Australia) for data management and analysis. Using a thematic analysis approach, transcripts were first read separately by both the lead investigator and a trained research assistant who identified, discussed and agreed on broad thematic tracks for initial coding. Following multiple re-reading of the transcripts, the lead investigator and research assistant separately undertook coding based on this initial coding framework, in addition to developing and discussing coding as new sub-categories of themes were identified. Notes taken during data collection were also transcribed and translated into English to contextualize thematic areas, and support data analysis and interpretation. Once the lead investigator and research assistant were confident that no new coding areas had emerged, two additional research assistants were recruited to support the analysis of final transcripts. The lead investigator took final responsibility to review and resolve variations in interpretation.

### Ethical considerations

Ethical approval for this study was obtained from the Malawi National Health Sciences Research Committee, (#1057) and the London School of Hygiene & Tropical Medicine Ethics Committee (#6432). The overall trial is registered with ClinicalTrials.gov, NCT02213211. Additional approval to conduct discussion groups with school children was obtained from the Malawi College of Medicine Research Ethics Committee (#1759). Permission to conduct the study was also received from local administrative offices and district education and health offices in Zomba. All participants invited to take part were given an information sheet in the local language (Chichewa) and provided written consent before participation. For school children, written assent was provided after their parent or guardian had consented first. All participants were able to indicate on the consent form if they agreed for discussions to be recorded, or to be quoted anonymously. During transcription, names and locations of participants were replaced with unique identifiers. Recordings and transcripts were stored on password-protected computers held by the lead investigator.

## Results

In total, eight FGDs and 20 IDIs were conducted. Five FGDs were carried out with adults (parents, guardians and teachers), and three FGDs conducted with school children who had recently sought care from an LTK dispenser.

### Social demographic characteristics of participants

Of the 32 children involved in the FGDs, the youngest child was 11 years old and 75% were aged between 11 and 15 years. An equal ratio of male to female children participated in the FGDs to address any potentially diverging views relating to treatment seeking behaviour, due to a priori observation that girls appeared to be seeking significantly more LTK consultations than boys. For the adult FGDs, 32 female and 28 male individuals participated. Half of the participants were aged between 31 and 40 years, and almost 40% of participants (predominantly teachers) had obtained tertiary education. The remaining participants (60%) (predominantly parents and guardians) were primarily subsistence farmers or engaged in small-scale business. The results of the FGDs and IDIs are presented by key themes. In general, the responses of participants (teachers, parents and guardians, and children) from schools with different levels of uptake did not significantly differ, and are therefore presented together.

### Understanding of the LTK by different stakeholders

Teachers, parents and guardians, school children, and healthcare workers as well as key stakeholders generally understood that the purpose of the LTK was to provide care for basic illnesses and injuries for children enrolled at that particular school during school time, rather than for other children or the wider community as a whole. The majority of community members were also aware that if a child became sick at home after the end of the school day, during weekends, or during the school holiday, it was still the responsibility of a parent or guardian to take the child to the health facility for treatment rather than to wait for the LTK to be available.

*“[The LTK] It is mainly concerning the treatment of learners when they are sick at school especially on the part of malaria; we have LTK dispensers who are doing that job, they were trained by Save the Children”.*

IDI with head teacher.

Teachers and education officials reported that children had developed a trusting relationship with the LTK dispensers in their capacity as both a healthcare provider as well as an educator, and that these roles were perceived as complementary rather than contradictory.

*“The learners trust us more than their class teachers. Sometimes when the learner is sick, quietly s/he leaves the class for LTK room without the knowledge of their class teacher. When you tell the learner to wait for you at break time so that you can treat them, they feel that you are taking long. They want to be treated right away. They view us as teachers and doctors at the same time.”*

LTK dispenser FGD.

However, it was frequently reported by teachers that parents and guardians had not initially understood the specific target and scope of the LTK, in addition to initial concerns arising from uncertainty in the teachers’ ability. Schools reported that they had commonly used community sensitization meetings to resolve these challenges, but also that once community members saw the programme in action it had been enthusiastically accepted and supported.

*“There was a day when my child was sick and I was passing by. Then I had to escort my child to the LTK room, I saw exactly what the dispenser did. I did not see any problem with testing and treatment of my child. We have confidence in the dispensers because they were trained, therefore if they say that my child has malaria, I believe the results.”*

FGD with parents and guardians.

Nevertheless, at times it was still difficult for parents and guardians to appreciate that the LTK was not an extension of the “village clinic”, community case management (CCM) or a component of the integrated management of childhood illnesses programme (IMCI) conducted by a salaried community health worker known as a Heath Surveillance Assistant (HSA). LTK dispensers reported that at times it was not only sick school going children who were brought to the school out of hours, but that parents and guardians themselves had also requested treatment.

*“Yes, people approach us, and plead with us, that we should assist them because the health facilities where they can be tested and treated for malaria are very far. They have heard that at school, we do have malaria test kits and treatment, therefore they request us to assist them but we refuse.”*

FGD, LTK dispenser.

### Benefits of the school-based malaria programme

A number of benefits of the LTK were reported by children, parents and guardians, and teachers. Teachers described that apart from acquiring additional skills with regard to malaria testing and treatment, school absenteeism and the number of school drop-outs had reduced. These perceived benefits to school attendance and performance, rather than directly to an outcome in terms of health, were also shared by district and national officials as well as by children themselves. School children, parents and guardians also highlighted the fact that the LTK had relieved some of the economic burden (both opportunity and financial cost) of taking a child to the health facility.

*“When you are sick, you do not have to be absent from school you still come; you go to the LTK room, receive medication and go back to class. Our parents are very happy because they are not having any problem with taking us to the health facility to receive treatment so they say they are able to save money.”*

School children FGD.

*“It is a great relief to us, instead of us parents taking the sick learner to the health facility for treatment, we are spared that task. The burden is lifted from us. The learner is assisted at school and attends classes.”*

FGD with parents and guardians.

District and national level key stakeholders reported that the LTK was consistent with the Malawi’s School Health and Nutrition (SHN) 2008–2018 strategic plan, which aims to have “healthy school-age children who can fulfil their optimum learning potential” [10]. They recognized that school children had not previously been addressed with specific malaria interventions, and that the LTK had raised greater awareness of the burden of malaria in this age group.

*“Yes, because that (school) is the contact point with children. You know previously we used to say that the most affected are the under*-*fives, but what we are seeing now, with this coming data: you find a nine years old, eight years, these are maybe in class 5, class 4, class 3, they are equally affected. So this study is an eye opener because it is like a shift from our previous belief that it is only the under*-*fives that we should focus on*—*those who are really greatly affected.”*

IDI with NMCP official.

Additionally, stakeholders also reported that LTK dispensers had been observed providing relevant behaviour change communication messages around malaria control while conducting a consultation with a child. This was perceived to be a public benefit of the LTK to the broader community, as children were acting as message carriers into the wider community.

*“There are several people that have received mosquito nets and they have misused. Since teachers are also emphasizing the use of bed nets, the child will pass on this information to the parents and we might see changes in bed net use. I think it is the right channel to pass this information.”*

IDI with official from MOEST.

Healthcare workers reported that the LTK had reduced workload at the local health facilities, since some of the children who would have sought treatment there were now being managed at school. Moreover, policy makers expressed strong support for the LTK, and expressed provision for scaling up should it prove feasible.

*“I think schools are the appropriate place for this mainly because it will reduce attendance at the health facilities, if we prevent these clients coming to the facilities, that means even the work load at the facility will be lessen.”*

IDI with official from MOEST.

### Perceived roles of different stakeholders

It was appreciated that while the roles and responsibilities of each stakeholder was different, they were broadly supporting and complementary. In particular, it was understood that teachers were now responsible to test and treat sick learners, while head teachers supported and supervised them. The MoH provided drugs and supplies, whilst the District Health Office supported teachers through supervision and mentorship. The MoEST and Save the Children Malawi provided technical support.

*“We supply the schools with the malaria kits, we can supervise, train and also involve our malaria focal person. So I think it starts from the supplies, supervision and review meetings and in future we can incorporate them into our programmes.”*

IDI, Senior District Health Official.

### Relationship between healthcare workers and LTK dispensers

One major barrier to implementation frequently raised by participants from both the education and health sectors, particularly LTK dispensers and healthcare workers, was that there had sometimes been poor working relationships between the two groups, especially in the early stages of implementation. This had ultimately led to a common perception amongst LTK dispensers that the LTK was of low priority to healthcare workers, most notably during the collection of drugs and supplies. However, some healthcare workers expressed that this was rather due to the fact that they had been inadequately orientated.

*“Initially we did not know what to do with the new programme and some teachers were sent back without supplies but after they included us in the training, teachers were supported accordingly.”*

IDI, healthcare worker.

LTK dispensers expressed an expectation that they should have been supervised by healthcare workers from the nearest health facility, but that this had rarely occurred in practice. This resulted in concerns that a lack of supervision could lead to a detrimental loss of both knowledge and skills.

*“Normally when you are doing an activity outside your area of expertise, you need to have mentors close. Teachers went to health facilities for mentorship but no health worker visited the schools as was required.”*

IDI with MOEST official.

In the event where an LTK dispenser was unable to assist a child and had referred them to a health centre, LTK dispensers were trained to fill a referral form that would be completed by the receiving healthcare worker and subsequently returned to the LTK dispenser once the child returned to school. However, LTK dispensers frequently reported that they did not always receive feedback from healthcare workers regarding the referred children, and that parents and guardians were often not given the referral form back by health workers after having sought care. This prevented LTK dispensers from knowing what, if any, action had been taken regarding the child’s original health complaint.

*‘The other problem we see is on referral, you find that our friends at the health facility, they do not give the mothers the feedback slips, we do not know whether the child we referred was assisted or not.”*

LTK dispenser FGD.

### Supply chain management and collection of supplies

At times where only limited supplies were available at the health facility, or where certain supplies were persistently stocked out, LTK dispensers often perceived this to be the result of health workers prioritizing the supply chain requirements of community case management of integrated childhood illnesses over the LTK. Recognizing the broader challenges experienced in effective supply management to the health sector, senior health officials expressed concern over maintaining the supply chain for the LTK without external support.

*“I think this programme at school level will be difficult to run…it is a major problem to keep the LTK supplies adequate because currently government supplies are meant for health facility or village clinics.”*

IDI with senior health officer.

In addition, LTK dispensers frequently raised problems regarding the availability, costs incurred or appropriateness of transport to collect supplies from the health facility. Some LTK dispensers reported incurring personal costs undertaking this task, and called for additional financial or in-kind (e.g., a bicycle) support.

*“We do use bicycles but there is a challenge in the sense that if the LTK dispenser does not have a bicycle, she or he has to borrow and in cases where the borrowed bicycle is broken, the LTK dispenser should mend it at his or her cost.”*

LTK dispenser FGD.

### Increased workload and demand for incentives

A common challenge regarding the LTK from the perspective of implementers was the high workload created by attending to sick children, especially during the peak malaria season when the number of children seeking care was relatively high, and a perceived lack of adequate compensation for the additional work. Teachers reported that the short duration of break time (usually fewer than 30 min) was often insufficient to attend to all the children seeking care, causing teachers to be delayed in returning to their teaching duties. While some reported that the head teacher would call in another teacher to attend to the class when this occurred, it was not necessarily effective as the substitute teacher had not prepared for the lesson, and would be limited to simply supervising the class.

*“We also assist by making learners quiet as they wait for their teacher who is at LTK room*.”

FGD with non-LTK dispensers.

*“I visited schools implementing this programme in the district and the challenge that I noticed is the workload. Teachers have to attend to their classes and the sick learners.”*

IDI with Senior Ministry of Education official.

*“LTK dispensers do not rest. We do not have time to rest. The moment you have gone to LTK room to treat learners you do not have time to rest because sick learners come one after the other especially from January to March when malaria cases are at a peak. Therefore, there is a huge workload it would be good if all teachers were trained to share the workload.”*

LTK dispensers FGD.

All stakeholders expressed a need to increase the number of LTK dispensers trained per school from an average of three up to six, depending on the total school enrolment. This was intended as a strategy to address conflict between the LTK dispensers and non-dispensers resulting from the perception by non-LTK dispensers that LTK dispensers were not fulfilling their core duty of teaching; but also to relieve LTK dispensers of their individual workloads and hence reduce some of the dissatisfaction that they were undertaking additional work without additional pay.

*“The number of trained teachers and the size of the school, that should be looked into critically otherwise they will be just treating pupils than teaching and that can create chaos in schools.”*

IDI with Senior District Health Official, Zomba.

Because of the perceived increase in workload, many stakeholders expressed a need for appropriate and adequate remuneration as crucial for the LTK to be sustained. This was most commonly framed as a motivation for teachers to undertake the role, but also as a justification that the treatment of children was an additional responsibility for a teacher and should therefore be compensated as an addition to their regular salary. The most commonly suggested methods of remuneration were financial, such as monthly allowances or review meetings and training.

*“My comment is that the programme planners should give dispensers incentives as you know that this work is very involving.”*

LTK dispensers FGD.

## Discussion

In the case of our study, malaria testing using RDTs and treatment using ACT by trained school teachers was well received and perceived to be a valuable programme amongst both providers and users of the LTK. While presumptive malaria diagnosis and treatment has been shown to be feasible and acceptable when delivered by teachers [11–13], this study is the first to show that the use of RDTs and ACT by teachers can also be an acceptable way of delivering care for malaria. This finding is significant for malaria control, because whilst access to health facilities is limited in most African countries including Malawi, enrolment at primary school is high in most rural communities [8]. This provides an opportunity for the health and education sectors to collaborate in improving the health and learning environment for school-going children, and to ensure timely access to prompt diagnosis and treatment, one of the key strategies in malaria control [17]. It is important to highlight that as school-based malaria testing and treatment can only be done during term time and school hours, school children could still delay in receiving prompt care and treatment when sick outside these times. Therefore, ensuring that all individuals suffering from malaria have prompt access to effective treatment remains a priority for resource-constrained health systems, regardless of the availability of programmes such as the LTK.

For a programme where teachers use RDTs and ACT to diagnose and treat uncomplicated malaria to be successful, there is need for an effective collaboration between the health and education sectors. Although it is clear that teachers can safely and accurately test and treat malaria in school children [16], supervision and mentoring remain critical elements in the delivery of any community-based programme and thus LTK dispensers will require technical, logistical and supply chain support from healthcare workers in local health facilities. However, concerns regarding poor coordination between the health and education sectors were shared by local, district and national stakeholders. Teachers expressed concerns that they regularly failed to receive feedback on referred patients, or that their requests for drugs from the local health facility were not prioritized. Such issues were observed in a project training shopkeepers to use RDTs and placing this service in drug shops in Uganda, where the links between the informal drug shops and formal health sector were also perceived to be weak [18]. Similar to these results, health workers reported not being fully oriented on the newly introduced programme, and shopkeepers reported referral forms given to clients were not formally recognized or respected by health workers.

During the trial, facility-based healthcare workers were trained and designated as local supervisors of teachers, although additional supervision visits were conducted by both the implementation team and district taskforce. However, teachers reported that supportive supervision and mentoring to the LTK dispensers by healthcare workers was limited. Recent research corroborates these accounts, reporting that deployment of facility-based healthcare workers as supervisors of community-based health services is frequently unrealistic and unsustainable [19]. Whilst a lack of supervision of peripheral services and inadequate referral systems are not unfamiliar challenges to many low-income country’s health systems, it is important that these processes continue to be strengthened if the LTK was to be rolled out more widely.

Although calls for greater supervision of peripheral health services are frequently reported, little consensus exists of the best approach, effectiveness, or affordability of such strategies [20]. For the LTK in Malawi, community-based Health Surveillance Assistants (HSAs) would likely be the best supervisors for teachers given their experience in providing health services at community level. HSAs are currently responsible for treating common childhood diseases, including malaria, in children under five in hard-to-reach areas [21] and as such have good experience in dealing with the formal health sector, coordinating an effective supply chain and biowaste disposal. However, given current concerns regarding the detrimental effects of ‘overloading’ HSAs in both their responsibilities and scope, this should be approached cautiously [21]. Whatever the approach, future roll-out of any school-based malaria test and treat programme can only be successful if efforts are made to ensure effective support and supervision of the teachers by local health workers.

Establishing a robust, reliable and consistent supply chain is critical for a community-based case management intervention such as the LTK, but is a frequently reported challenge to such interventions [22]. In Malawi, more than one-third of all HSAs surveyed did not have all essential drugs in stock, and the majority of clinical errors were due to not having the required treatments. In this study, supplies such as ACT, RDTs and other LTK supplies were procured by the implementing partner and delivered to schools from local health centres through the district health office, limiting stock-outs in the study area because of this parallel supply provided for the study. Stock-outs are the most commonly reported obstacle to providing services to HSAs, followed by delays between requesting and receiving supplies and then transport-related obstacles [20], issues that were also raised by the LTK dispensers in this study. Ongoing disruption and stock-outs of essential drugs in Malawi [21] has significant implications for the entire health system, including a routine school-based programme where supplies would have to be integrated with the general supply. The unavailability of drugs, including at health facilities, was also reflected in concerns from school children, who reported that drugs meant for unwell school children were sometimes shared with other individuals. While strengthening the LTK supply chain at the *last*-*mile* is, therefore, a prerequisite of successful implementation, it must take place with attention to and in broader support of the entire supply chain system.

Due to the unexpected high levels of demand for the LTK, trained teachers and their head teachers described being put under significant strain, and that their time spent teaching was affected as they could not leave sick children unattended. The two biggest implications of this are overburdening of teachers and a negative impact on learning for school children. To address this concern, such a programme could train a greater number of teachers per school to reduce the workload on individual teachers and to minimize any disruption to teaching. In addition, LTK dispensers expressed an expectation that because they were doing an extra activity on top of teaching, they should be rewarded with an incentive. This finding is similar to what others have shown for school-based malaria programmes [14]. Whilst incentives have been shown to motivate, retain and increase the participation of workers [23] such an incentive would have to be thought of in the context of other school-based health programmes. Currently, programmes such as school feeding or routine deworming are generally facilitated by teachers without an incentive. Introduction of an incentive for a school-based malaria programme could potentially disrupt the success of other programmes, if the incentive is seen to be targeting only teachers working on the malaria programme.

There are several limitations to be considered when interpreting results from this study. The implementing partner has a long established and recognized presence in the study area, implementing child health programmes in support of government efforts. At the same time, the study was conducted at a time when the health facilities in Malawi faced critical shortages of drugs and supplies. In this environment, participants may have expressed their acceptability of the LTK programme from the perspective that schools are the only viable option for health for their children and for fear of jeopardizing future support from Save the Children.

The scope of this nested study was to explore the experiences and perceptions of those who had either sought, delivered or supervised the provision of care by school teachers at the school level, and thus all those who participated in the study had direct experience in the implementation of the LTK. Parents, guardians and school children were selected on the basis of recent (less than 6 weeks since) treatment, representing a specific population of all stakeholders who may not represent the broader views of those who have never required care from the LTK, or never chosen to seek care from the LTK. In addition, only children from senior classes (Standard 6–8) were involved in this study, who represent both the smallest overall enrolled population and the age group with the least number of total LTK consultations, and thus do not necessarily represent the opinions of the majority of children who ultimately seek treatment from the LTK. Finally, the study involved only community members from intervention schools, and did not include the views of parents, guardians, or children from schools not receiving the intervention.

Awareness of broader perspectives, including the acceptability of the intervention in naïve as well as non-participant community members would have reduced the chance of positive bias in the reported perceptions of the intervention. Including these groups in future qualitative research could also contribute important insights on pre-intervention expectations and concerns that could be prospectively addressed during early implementation activities such as community sensitization, training or supervision. Indeed, observations that a proportion of school children in intervention schools continued to seek care from other formal and informal sources despite the LTK being available (Halliday et al. submitted) should be a priority focus for future research regarding how the intervention is perceived by these individuals, and thus how the LTK and similar school-level health interventions could be refined.

## Conclusions

Overall, the LTK was perceived by users and implementers to bean acceptable method of delivering care for malaria to school children by parents and guardians, teachers and district officers alike. For many, the LTK was considered a preferential choice for sick children due to its perceived convenience and reliability in terms of facilitating prompt treatment, saving time and money and allowing children to continue with their education. However, the perceived benefits of the programme created an unexpectedly high demand for the service, which led to concerns from providers such as maintaining the supply chain from local health facilities, overburden of additional work for teachers, and requests for treatment for ineligible community members at inappropriate hours. With careful consideration to appropriate capacity building, redeployment of resources and integration of services at school level to ensure sustainability and impact, there was strong support to scale-up the programme, and further evaluation of such scale-up is warranted.
